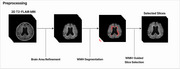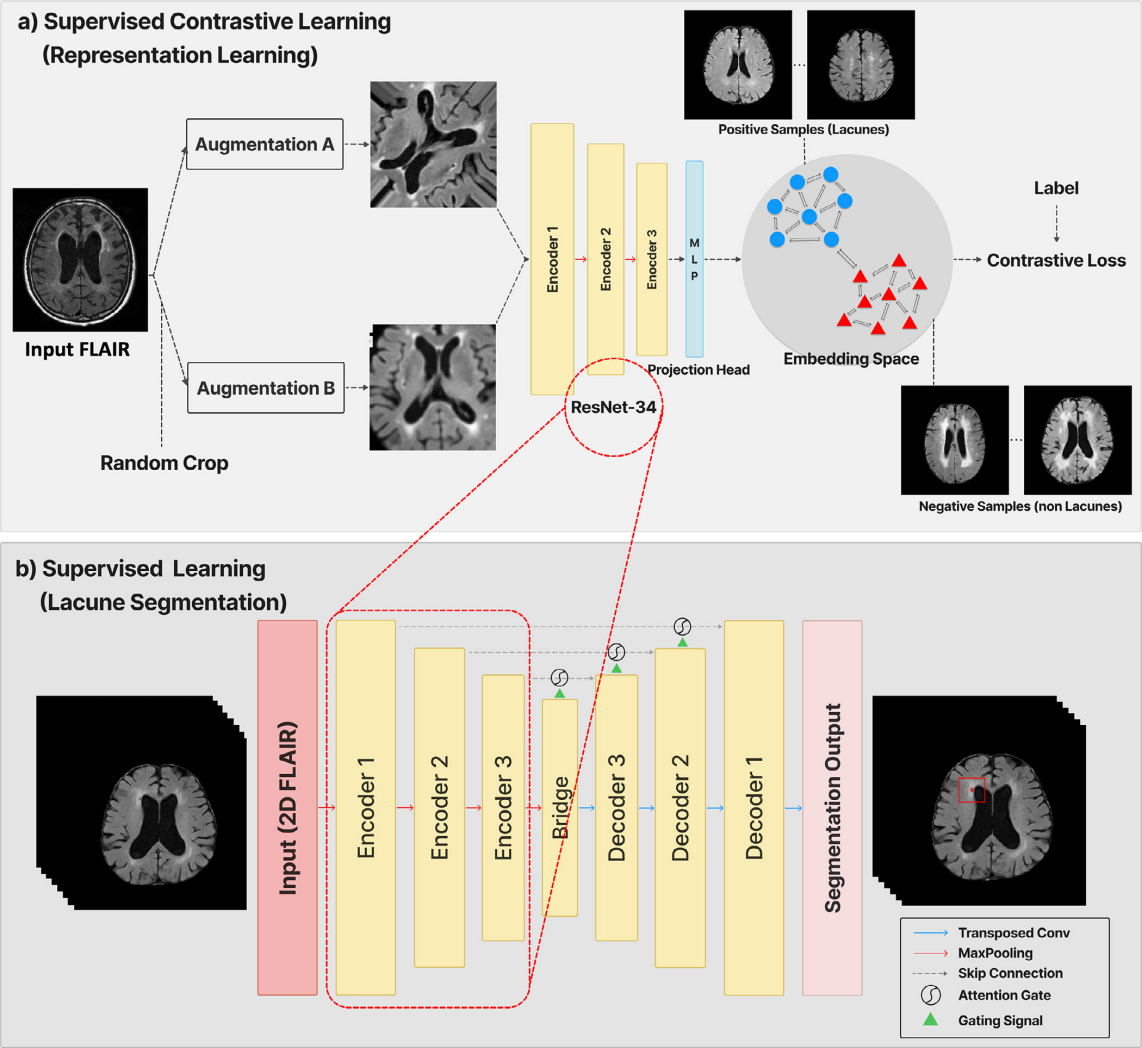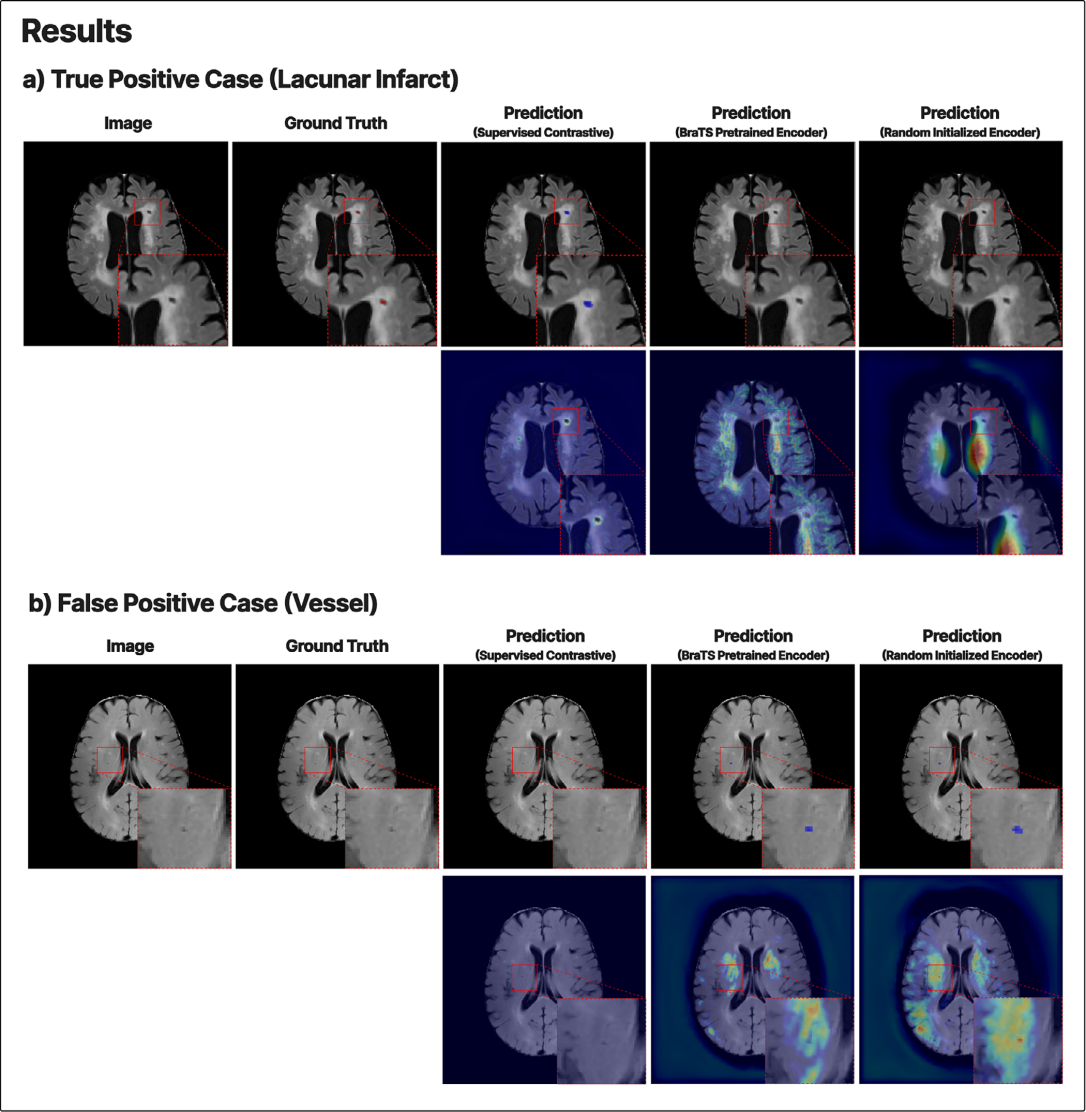# Discriminative Feature Learning for Lacune Detection in 2D T2‐FLAIR Images using Supervised Contrastive Learning

**DOI:** 10.1002/alz70862_109968

**Published:** 2025-12-23

**Authors:** Minwoo Han, Saehyun Kim, Wooseok Jung, Seung Hyun Lee, Chong Hyun Suh

**Affiliations:** ^1^ University of Ulsan College of Medicine, Seoul, Seoul Korea, Republic of (South); ^2^ VUNO Inc., Seoul, Seoul Korea, Republic of (South); ^3^ VUNO Inc., Seocho‐gu, Seoul Korea, Republic of (South); ^4^ Asan Medical Center, University of Ulsan College of Medicine, Seoul Korea, Republic of (South)

## Abstract

**Background:**

Lacunes (of presumed vascular origin) are associated with an increased risk of cognitive decline and may elevate the risk of amyloid‐related imaging abnormalities (ARIA). This study aimed to develop a deep learning‐based model for automatic segmentation of lacunes that can effectively distinguish them from lacune‐mimicking features in an imbalanced dataset.

**Method:**

A total of 427 T2‐FLAIR MRI images from Asan Medical Center were used for model development. One neuroradiologist manually segmented all cases of lacunes to establish ground truth. The dataset was divided into training, validation and test sets with a 3:1:1 ratio. Preprocessing of all MRI images included intracranial volume (ICV) segmentation and white matter hyperintensity (WMH) segmentation using VUNO Med‐DeepBrain (validation DSC, 0.988, 0.855, respectively). Attention U‐Net architecture with a pretrained encoder using supervised contrastive learning was developed. Predictions with centers within 3mm of ground truth centers were regarded as true positives. The diagnostic accuracy of the model for instance‐level detection of lacunes were evaluated using alternative free‐response receiver operating characteristics (AFROC) analysis. Additionally, subgroup analysis to assess patient‐level outcomes were performed, including: (1) detection of the presence or absence of lacunes, (2) discrimination between no vs 1–2 lacunes, and (3) discrimination of no or 1–2 lacunes vs lacunes of 3 or more.

**Result:**

The test set included 55 positive patients with a total of 166 lacunes, and 31 negative patients without lacunes. For instance‐level detection, the model correctly identified 102 out of 166 lacunes (61.5%; 95% CI, 51.2–70.8%) with a figure‐of‐merit (FOM) of 0.726 (95% CI, 0.635–0.816). At the patient‐level, the AUC for lacune detection was 0.810 (95% CI, 0.724–0.896). The model achieved a sensitivity of 58.1% (95% CI, 40.8–73.6%) in identifying patients with 1–2 lacunes and 58.3% (95% CI, 38.8–75.5%) in identifying patients with lacunes of 3 or more.

**Conclusion:**

This study introduces a deep‐learning based approach for detecting lacunes from highly imbalanced dataset using encoder pretrained with supervised contrastive learning. Future work will focus on expanding the current approach to include lacune localization within specific brain regions and conducting external validation across multiple centers.